# Mitochondrial alterations in pellagra-associated central chromatolysis: Comparison with ballooned achromatic neurons of other etiologies

**DOI:** 10.1093/jnen/nlag058

**Published:** 2026-08-06

**Authors:** Araki Kimura, Ito Kawakami, Kenji Ikeda, Akito Nagakura, Kenichi Oshima, Tadafumi Kato, Masato Hasegawa

**Affiliations:** Department of Clinical Medical Sciences, Dementia Research Project, Tokyo Metropolitan Institute of Medical Science, Setagaya, Tokyo, Japan; Department of Psychiatry & Behavioral Science, Juntendo University, Bunkyo, Tokyo, Japan; Department of Psychiatry, Tokyo Metropolitan Matsuzawa Hospital, Setagaya, Tokyo, Japan; Department of Psychiatry, Tokyo Metropolitan Matsuzawa Hospital, Setagaya, Tokyo, Japan; Division of Molecular Pathology and Histology, Tokyo Metropolitan Institute of Medical Science, Setagaya, Tokyo, Japan; Department of Clinical Medical Sciences, Dementia Research Project, Tokyo Metropolitan Institute of Medical Science, Setagaya, Tokyo, Japan; Department of Psychiatry, Tokyo Metropolitan Matsuzawa Hospital, Setagaya, Tokyo, Japan; Department of Psychiatry & Behavioral Science, Juntendo University, Bunkyo, Tokyo, Japan; Department of Psychiatry, Tokyo Metropolitan Matsuzawa Hospital, Setagaya, Tokyo, Japan; Department of Psychiatry, Tokyo Metropolitan Matsuzawa Hospital, Setagaya, Tokyo, Japan; Department of Psychiatry & Behavioral Science, Juntendo University, Bunkyo, Tokyo, Japan; Department of Clinical Medical Sciences, Dementia Research Project, Tokyo Metropolitan Institute of Medical Science, Setagaya, Tokyo, Japan

**Keywords:** central chromatolysis, mitochondria, neuropathology, niacin, pellagra

## Abstract

Neuronal central chromatolysis (CC) is the histopathological hallmark of pellagra encephalopathy, a neurological deficit resulting from vitamin deficiencies. Pellagrous CC neurons are morphologically similar to ballooned achromatic neurons in other conditions but the distinct pathomechanisms remain unclear. We performed a clinico-neuropathological analysis of 10 autopsy cases of pellagra encephalopathy. The pellagra encephalopathy cases were immunohistochemically compared with disease controls, including cases of axonal injury and neurodegenerative diseases. Electron microscopic evaluation and immunohistochemical examinations targeting mitochondrial fragmentation were performed for a representative case. Four of 10 pellagra encephalopathy patients exhibited prolonged impairment of consciousness distinguishable from alcohol withdrawal delirium. Pellagrous CC neurons were negative for cytoskeletal markers whereas ballooned achromatic neurons in the disease control cases were positive. Immunohistochemical analysis of mitochondrial markers revealed that CC neurons exhibited more intense immunoreactivity for COX-IV and mitochondrial fissure factor compared to the disease controls. Transmission electron microscopy of these CC neurons revealed a marked increase in the mitochondria with amorphous densities. These findings indicate that the pathomechanism of pellagrous CC is distinct from that of the ballooned achromatic neurons of other etiologies. Mitochondrial alterations in pellagrous CC neurons suggest that neuronal energy deficits resulting from nicotinamide adenine dinucleotide deficiency induce mitochondrial fragmentation.

## Introduction

Pellagra is caused by deficiencies in vitamin B3 (niacin), B6 (pyridoxal), and tryptophan. Clinically, it is characterized by the “4Ds”: gastrointestinal symptoms such as diarrhea, dermatitis, dementia, and (if untreated) death.^1^ Pellagra encephalopathy is a neurological manifestation consisting of disturbances of consciousness, hallucinations, delusions, and convulsions in addition to dementia.^2^^,^^3^ Although pellagra is relatively infrequent in developed countries, it can be caused by low nutritional status, particularly chronic alcohol abuse,^2–4^ anorexia nervosa,^5^^,^^6^ and corn diet.^1^ Pellagra can also be caused by certain medications, such as azathioprine,^7^ which inhibits the synthetic pathway of nicotinamide adenine dinucleotide (NAD^+^) from tryptophan, and isoniazid,^8^^,^^9^ which is a vitamin B3 analogue that reduces the vitamin B6 required for NAD^+^ synthesis.

Alcohol dependence syndrome, a common background factor for pellagra in developed countries, is associated with alcohol withdrawal symptoms.^10^ Alcohol withdrawal delirium after drinking cessation usually recovers within a few days with appropriate treatment.^11^ In contrast, pellagra encephalopathy is a potentially fatal disease if left untreated and has important clinical significance even today in terms of its possible involvement in prolonged consciousness disturbances in cases of alcohol use disorder. To more effectively differentiate alcohol withdrawal delirium from pellagra encephalopathy presenting with impaired consciousness, a thorough understanding of the pathomechanisms of pellagra encephalopathy is essential. Such knowledge would elucidate why specific lesions develop in characteristic locations within the central nervous system.

The pathophysiological mechanisms of pellagra encephalopathy are postulated to involve abnormalities in several pathways, including the NAD^+^ synthesis pathway, the tryptophan-kynurenic acid pathway, the mitochondrial ATP generation related pathway, the Poly (ADP–ribose) polymerase (PARP) pathway, the BDNF–TrkB axis, and a genetic background with vulnerability to niacin deficiency.^12^ However, much remains unknown. One of the reasons why the pathogenesis of pellagra encephalopathy is not fully understood is the difficulty in establishing animal models of pellagra encephalopathy. This difficulty arises mainly because laboratory mice and rats can synthesize NAD^+^ from tryptophan dozens of times more efficiently than humans.^13^ Although a few animal models of pellagra have been reported,^14^^,^^15^ to the best of our knowledge, none has specifically replicated pellagra encephalopathy. Consequently, to elucidate the pathomechanism of pellagra encephalopathy, histopathological studies of human patients are essential.

In pellagra encephalopathy, NAD^+^ deficiency is presumed to occur. NAD^+^ deficiency also occurs in other brain injuries, such as cerebral infarction,^16^ where mitochondrial fragmentation is observed in histological sections.^17^ Mitochondrial fragmentation is a cellular stress response through mitochondrial fissure factor (Mff)-mediated mitochondrial fission.^18^^,^^19^ Mitochondrial fragmentation has also been observed with agents that cause mitochondrial depolarization in cultured cells, a process that is known to be accompanied by changes in cristae structure and the dynamics of dynamin-like 120k-Da protein (OPA1), which is required for its maintenance.^20–23^ However, the involvement of mitochondrial fragmentation in pellagra encephalopathy remains unclear.

Pellagra encephalopathy is known histopathologically for central chromatolysis (CC), which is typically observed in pontine nuclei and other brainstem nuclei. Pellagrous CC is a neuronal histopathological finding characterized by nuclear marginalization, chromatin displacement, and cell body swelling.^24^ Morphologically similar findings, often referred to as achromatic or ballooned neurons (BN), are observed in retrograde changes following axonal injury,^25^ and in neurodegenerative diseases such as corticobasal degeneration (CBD)^26^ and Pick disease (PiD).^27^ Currently, clear criteria to differentiate these morphologically similar findings are not well established. The differences in the mechanisms of lesion formation under each of these conditions with similar histopathological features also remain unclear. In particular, the potential involvement of NAD^+^ deficiency and mitochondrial fragmentation in the development of pellagrous CC remains unknown.

In this study, we present a detailed clinico-neuropathological study of pellagra CC cells, from the autopsy brains of patients with pellagra encephalopathy. The aims of this study were to elucidate the neuropathological evidence supporting an association between NAD^+^ deficiency and pellagrous CC, and to differentiate pellagrous CC from ballooned achromatic neurons of other etiologies.

## Methods

### Participants

Ten cases of pellagra encephalopathy were registered in the autopsy archives of Tokyo Metropolitan Institute of Medical Science and Tokyo Metropolitan Matsuzawa Hospital (Table 1). Cases of pellagra encephalopathy were defined as those with a clinical history of a nutritional disorders and neuropathological findings of CC cells in the central nervous system. Each case of pellagra encephalopathy was retrospectively reviewed via patient clinical records. For comparison, immunohistochemical findings of 1 axonal injury case, 3 CBD cases, and 3 PiD cases, all of which exhibited BN, were analyzed and compared with those of pellagrous CC. Three out of 10 pellagra encephalopathy cases have been previously reported in a clinico-pathological study.^28^

**Table 1:** Clinical and demographic data of 10 patients with pellagra encephalopathy.

Case no.	1	2	3	4	5	6	7	8	9	10
**Sex**	M	M	M	M	M	F	M	M	F	M
**Age (years)**	64	59	55	71	54	72	63	57	Unknown	72
**Clinical diagnosis**	Alcohol use disorder, epilepsy	Alcohol use disorder	Alcohol use disorder	Alcohol use disorder	Alcohol use disorder	bvFTD	bvFTD	Epilepsy	Epilepsy, intellectual disability	Schizophrenia
**Prolonged disturbance of consciousness**	+	+	+	+	−	Difficult to evaluate	Difficult to evaluate	−	−	−
**Pellagra related clinical symptoms**	Diarrhea, skin pigmentation	−	−	Hypophagia, diarrhea	Diarrhea, nausea	Hypophagia, nausea, dermatitis	Hypophagia	Hypophagia, nausea, diarrhea	−	Hypophagia
**Nutritional administration status**	3 months of nasogastric tube nutrition	Reduced food intake due to alcohol disorder	Reduced food intake due to alcohol disorder	Reduced food intake due to alcohol disorder	Reduced food intake due to alcohol disorder	Several years of nasogastric tube nutrition	A year of nasogastric tube nutrition	Severe constipation and insufficient food intake	A month of central venous nutrition	6 months of central venous nutrition
**Comorbid neuropathological diagnosis**	−	−	Subdural hematoma	Obsolete infarction	Obsolete infarction	FTLD-TDP	FTLD-TDP	Obsolete infarction	Cerebellar atrophy	Obsolete infarction

Clinical and demographic data of 10 patients with pellagra encephalopathy. Seven patients presented with some of the classic “4Ds” symptoms of pellagra: gastrointestinal symptoms, dermatitis, dementia, and, in severe cases, death. Four patients had a clinical history of prolonged disturbance of consciousness.

Abbreviations: bvFTD, behavioral variant frontotemporal dementia.

### Histology and immunohistochemistry

Brain tissue was fixed in 10% formaldehyde and embedded in paraffin. Sections (8-μm thick) were cut from the hippocampus, precentral gyrus, superior frontal cortex, orbitofrontal cortex, midbrain, pons, and medulla oblongata (Table 2). The sections were stained with hematoxylin and eosin (H&E) and Klüver-Barrera (KB) stains. Immunohistochemical examination was performed using the following primary antibodies: anti-phosphorylated neurofilament (SMI-31; monoclonal, BioLegend), anti-non-phosphorylated neurofilament (SMI-32; monoclonal, BioLegend), anti-α-crystallin Β chain (αΒ-crystallin; monoclonal, Stress Marq), anti-phosphorylated tau (AT8 [ps202, pt205]; monoclonal, Invitrogen), anti-3-repeat tau (RD3; monoclonal, Sigma–Aldrich), anti-4-repeat tau (RD4; monoclonal, Sigma–Aldrich), anti-α-synuclein (pSyn#64 [ps129]; monoclonal, Abcam), anti-phosphorylated TDP-43 (ps409-410; monoclonal, Cosmo Bio), anti-glial fibrillary acidic protein (GFAP; monoclonal, PROGEN), anti-cytochrome c oxidase (COX-IV; monoclonal, Cell Signaling), anti-Mff ( polyclonal, Proteintech), and anti-dynamin-like 120k-Da protein (OPA1; polyclonal, Proteintech). Primary antibody labeling was visualized using DAB horseradish peroxidase polymer detection kits (Nichirei Biosciences). The specific brain regions examined by immunohistochemistry for each case are detailed in Table 3.

**Table 2:** Distributions and severity of central chromatolysis in 10 cases of pellagra encephalopathy.

Case no.	1	2	3	4	5	6	7	8	9	10
**Precentral gyrus**	1	3	3	3	2	1	2	2	0	2
**Oculomotor nucleus**	3	2	–	1	2	2	3	1	–	–
**Substantia nigra**	0	0	0	0	0	0	0	0	0	0
**Locus coeruleus**	0	0	0	0	0	0	0	0	0	0
**Raphe nucleus**	1	1	3	0	1	2	2	2	3	1
**Pontin nucleus**	0	3	3	3	3	3	3	3	3	2
**Pedunculopontine nucleus**	0	3	3	3	2	0	3	0	2	0
**Hypoglossal nucleus**	0	0	0	0	0	–	0	0	0	0
**Dorsal nucleus of vagus nerve**	0	1	2	1	2	1	0	2	2	2
**Cuneate nucleus**	2	2	3	3	3	2	–	2	1	2
**Gracile nucleus**	2	3	3	–	2	2	–	–	0	–
**Trigeminal spinal nucleus**	2	2	3	2	2	3	2	2	1	2
**Inferior olive nucleus**	0	0	0	0	0	0	0	0	0	0
**Reticular formation of pons and medulla oblongata**	1	3	3	3	2	2	2	1	–	1
**Arcuate nucleus**	0	2	2	3	2	2	–	3	0	2

Distribution of central chromatolysis (CC) cells in 10 cases of pellagra encephalopathy.

Abbreviations: 3, severe (almost all cells are chromatolytic); 2, moderate (obvious chromatolytic cells are present); 1, slightly (a few cells are suspected to be chromatolytic); 0, absent; (–), not assessed.

**Table 3:** Immunohistochemical findings in central chromatolysis neurons and ballooned achromatic neurons.

	Pellagra										Axonal injury	CBD			PiD		
	(with alcohol disorder)					(without alcohol disorder)											
Case no.	1	2	3	4	5	6	7	8	9	10	11	12	13	14	15	16	17
**Evaluated region**	ON	PN	PN	PN	PN	ON	PN	PN	PN	PN	OFG	SFG	SFG	SFG	CA	CA	CA
**Antibodies**																	
**SMI-31**	0	0	0	0	0	0	0	0	0	0	2	2	2	2	0	1	2
**SMI-32**	1	1	1	1	1	1	1	1	1	1	2	2	2	2	–	2	2
**αΒ-crystallin**	0	0	0	0	0	0	0	0	0	0	2	2	2	2	–	2	2
**AT8**	0	0	0	0	0	0	0	0	0	0	0	1	1	1	2	2	2
**RD3**	0	0	0	0	0	0	0	0	0	0	0	0	0	0	2	2	2
**RD4**	0	0	0	0	0	0	0	0	0	0	0	1	1	1	0	0	0
**COX-IV**	2	2	2	2	2	2	2	2	2	2	1	1	1	1	–	1	1
**Mff**	2	2	2	2	2	2	2	2	2	2	1	2	1	1	–	0	1
**OPA1**	0	1	0	1	1	1	1	1	1	1	1	1	1	1	–	0	0

Immunohistochemical findings in affected neurons from cases of pellagra encephalopathy, axonal injury, CBD, and PiD. Staining intensity in the affected neurons was graded on the following scale: 2; strongly positive. 1; weakly positive. 0; negative. (–); not done.

Abbreviations: CA, hippocampus; CBD, corticobasal degeneration; Mff, mitochondrial fissure factor; OFG, orbitofrontal gyrus; ON, oculomotor nucleus; PiD, Pick disease; PN; pontine nucleus; SFG, superior frontal gyrus.

### DAB staining optical density analysis and statistical methods

Statistical comparisons of the optical density of DAB staining were performed between the CC from 5 cases of alcohol use disorder-associated pellagra encephalopathy (Cases 1 to 5) and the BN from 4 cases of retrograde degeneration (Cases 11 to 14). The regions of interest for optical density measurement were delineated using Fiji (ImageJ version 1.54f),^29^ focusing on the cytoplasm of the swollen cells. The anatomical location of the cells targeted for optical density measurement corresponds to the regions evaluated for mitochondrial markers (Table 3). Statistical analysis was conducted using the Mann–Whitney *U* test in R (version 4.4.3; https://www.R-project.org/).

### Transmission electron microscopic examination

Brain tissue, which was fixed in 10% formaldehyde (Case 1; midbrain, Cases 2 to 5; pons), was refixed with 1% osmium tetroxide and embedded in resin. Ultrathin sections (70-nm thick) were cut from the lesions in the oculomotor nucleus. Light microscopic observation and identification of CC cells in sequential sections were performed prior to the preparation of ultrathin sections. The prepared sections were analyzed by transmission electron microscopy (JEM-1400; JEOL).

## Results

### Clinical findings in pellagra encephalopathy

The 10 pellagra encephalopathy patients included 5 with alcohol use disorders (Cases 1 to 5), 2 with neurodegenerative diseases (frontotemporal lobar degeneration with TAR DNA-binding protein 43; FTLD-TDP) (Cases 6 and 7), and 3 with epilepsy (Cases 1, 8, and 9) (Table 1). One patient (Case 1) was receiving treatment with antitubercular medications, including isoniazid. Seven patients exhibited gastrointestinal symptoms (Cases 1, 4, 5, 6, 7, 8, and 10); 2 exhibited skin disorders (Cases 1, 5). Prolonged disturbance of consciousness was observed in 4 patients (Cases 1, 2, 3, 4). None of the patients presented with all of the classic “4Ds.” Regarding nutritional status, 5 patients (Cases 1, 5, 6, 8, and 9) had received nasogastric or central venous nutrition for several months or longer. Four patients (Cases 2 to 5) had a markedly reduced food intake due to severe alcohol use disorder, and one patient (Case 9) had severe constipation and chronically reduced food intake.

### Distribution of CC cells in pellagra encephalopathy

**Figure 1:**
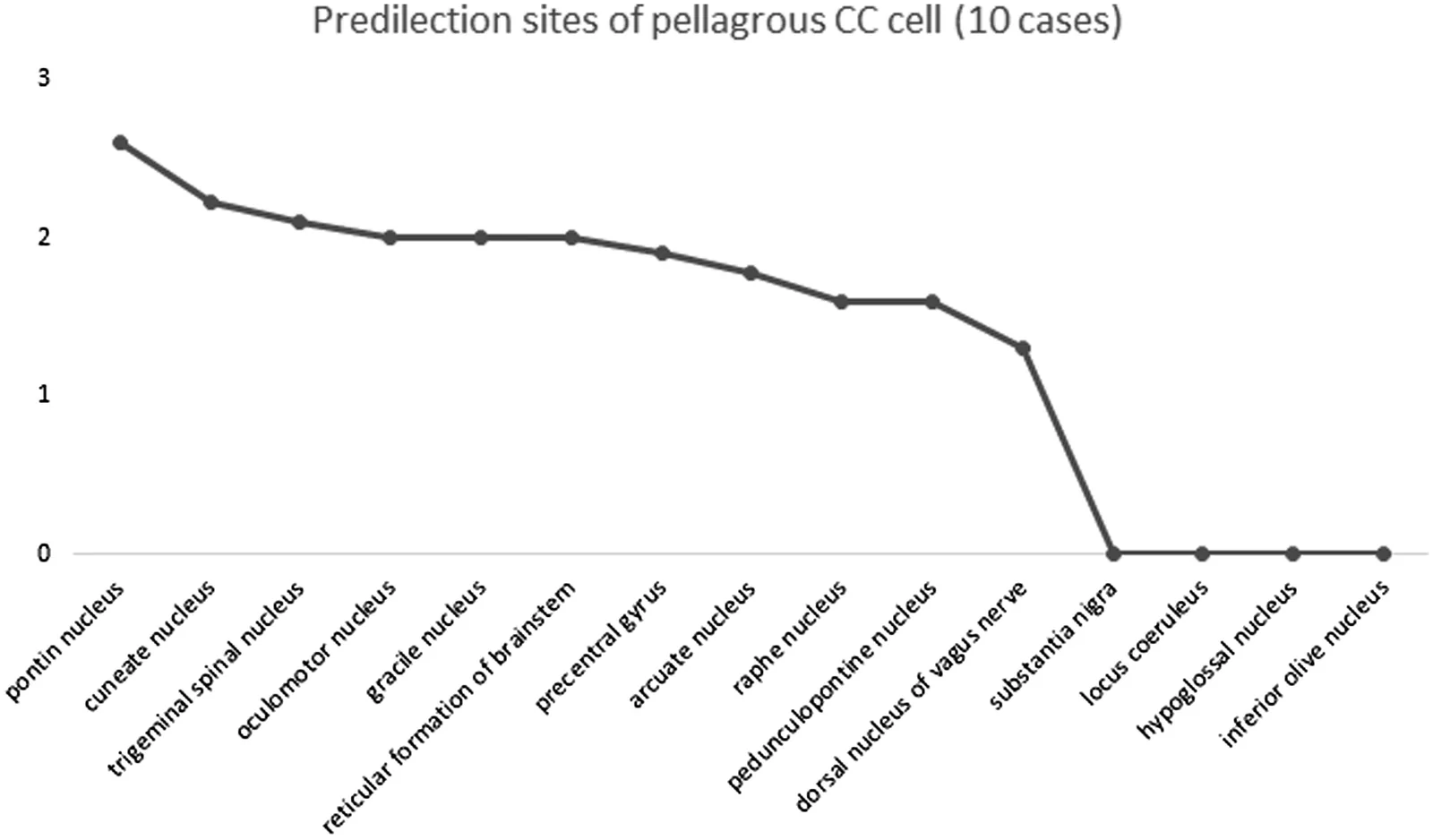
Predilection sites of pellagra-associated central chromatolysis (CC). This graph shows a representation of the mean severity scores for CC across various anatomical regions in the 10 pellagra encephalopathy cases. The CC cell distribution in each pellagra case is shown in Table 2. Severity scores: 3, severe (almost all neurons observed are chromatolytic cells); 2, moderate (obvious chromatolytic cells found in the observed area); 1, several neurons suspected to be chromatolytic cells; 0, absent.

CC cells were frequently observed in the precentral gyrus, oculomotor nucleus, raphe nucleus, pontine nucleus, pedunculopontine nucleus, trigeminal spinal nucleus, cuneate nucleus, gracile nucleus, arcuate nucleus, and reticular formation of the pons and medulla oblongata in 10 cases of pellagra encephalopathy (Table 2, Figure 1). No CC cells were found in the hippocampus, anterior cingulate cortex, or superior frontal cortex.

### Immunohistochemical findings in CC cells

**Figure 2:**
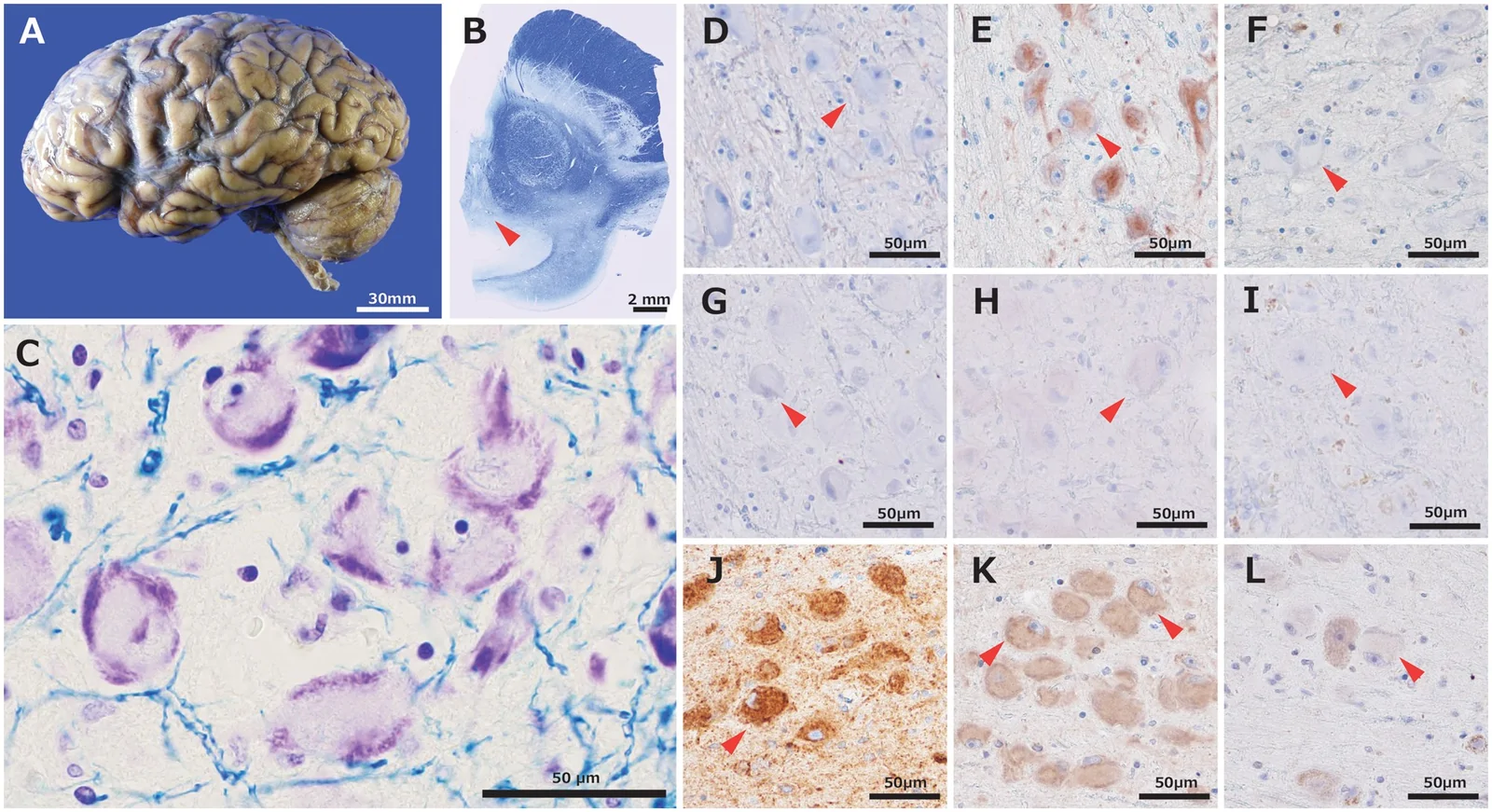
Neuropathological findings in a representative case of pellagra encephalopathy (Case 1). (A) Macroscopic findings. There is no evident cerebral or cerebellar atrophy. (B) Semi-macroscopic image of the midbrain (KB stain). The red arrow indicates an area where central chromatolytic cells are clustered. (C) Cell bodies were swollen; the chromatin and nucleus are marginalized (KB stain). (D-L) Immunohistochemical findings. (D) SMI-31, (E) SMI-32, (F) αΒ-crystallin, (G) AT8, (H) RD3, (I) RD4, (J) COX-IV, (K) Mff: staining for Mff in central chromatolysis (CC) neurons was preserved and comparable to that in non-chromatolytic neurons. (L) OPA1: staining for OPA1 in CC neurons was markedly reduced. The CC neurons were negative for cytoskeletal makers.

**Figure 3:**
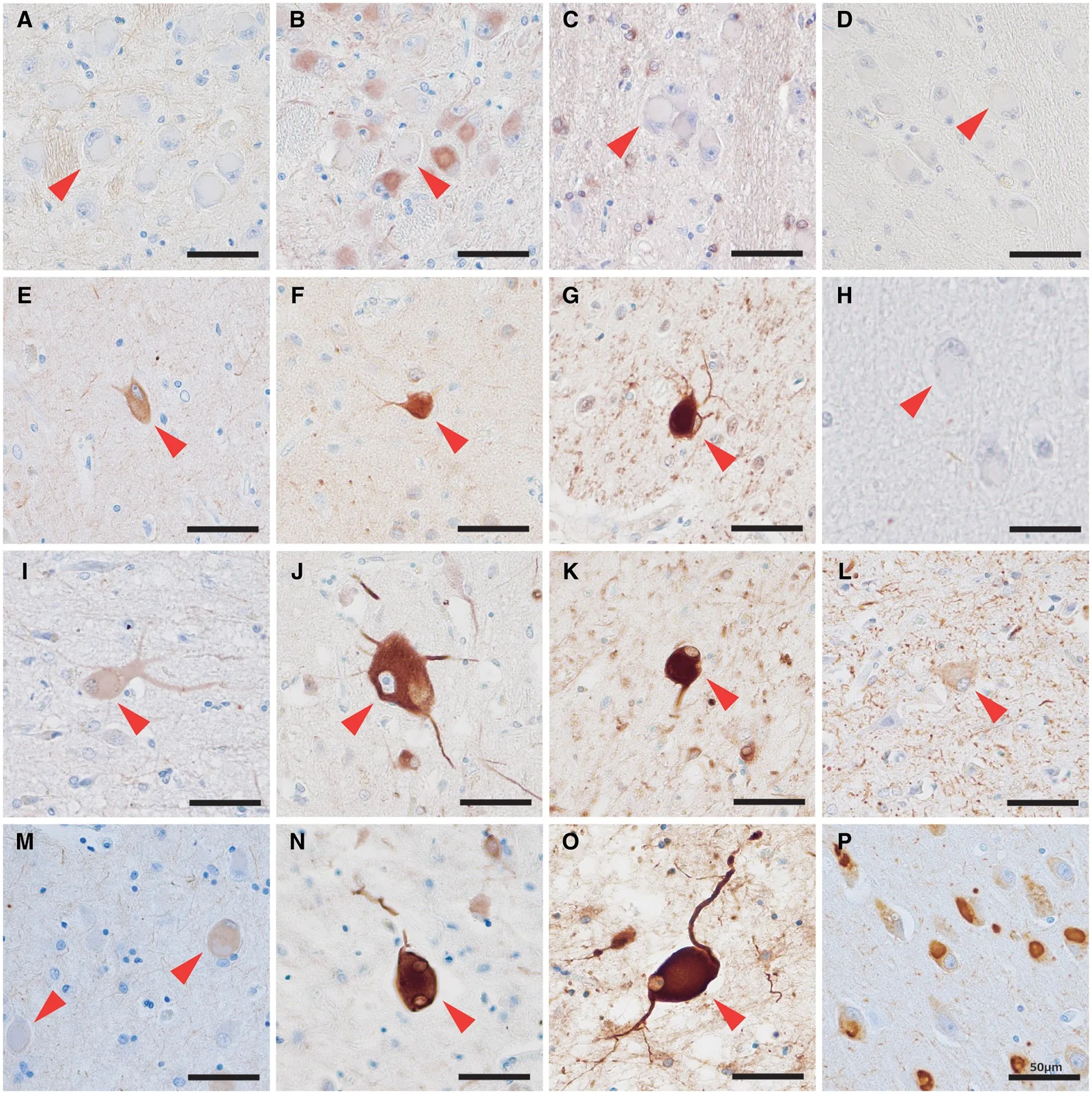
Immunohistochemical findings of cytoskeletal markers in central chromatolytic neurons from pellagra encephalopathy and ballooned achromatic neurons from disease control pathologies. Representative images of central chromatolytic neurons from pellagra encephalopathy cases and ballooned achromatic neurons from disease control cases. Pellagra encephalopathy (Case 2): (A) SMI-31, (B) SMI-32, (C) αΒ-crystallin, (D) AT8. Axonal injury (Case 11): (E) SMI-31, (F) SMI-32, (G) αΒ-crystallin, (H) AT8. Corticobasal degeneration (CBD) (Case 14): (I) SMI-31, (J) SMI-32, (K) αΒ-crystallin, (L) AT8. Pick disease (PiD) (Case 16): (M) SMI-31, (N) SMI-32, (O) αΒ-crystallin. PiD (Case 15): (P) AT8.

**Figure 4:**
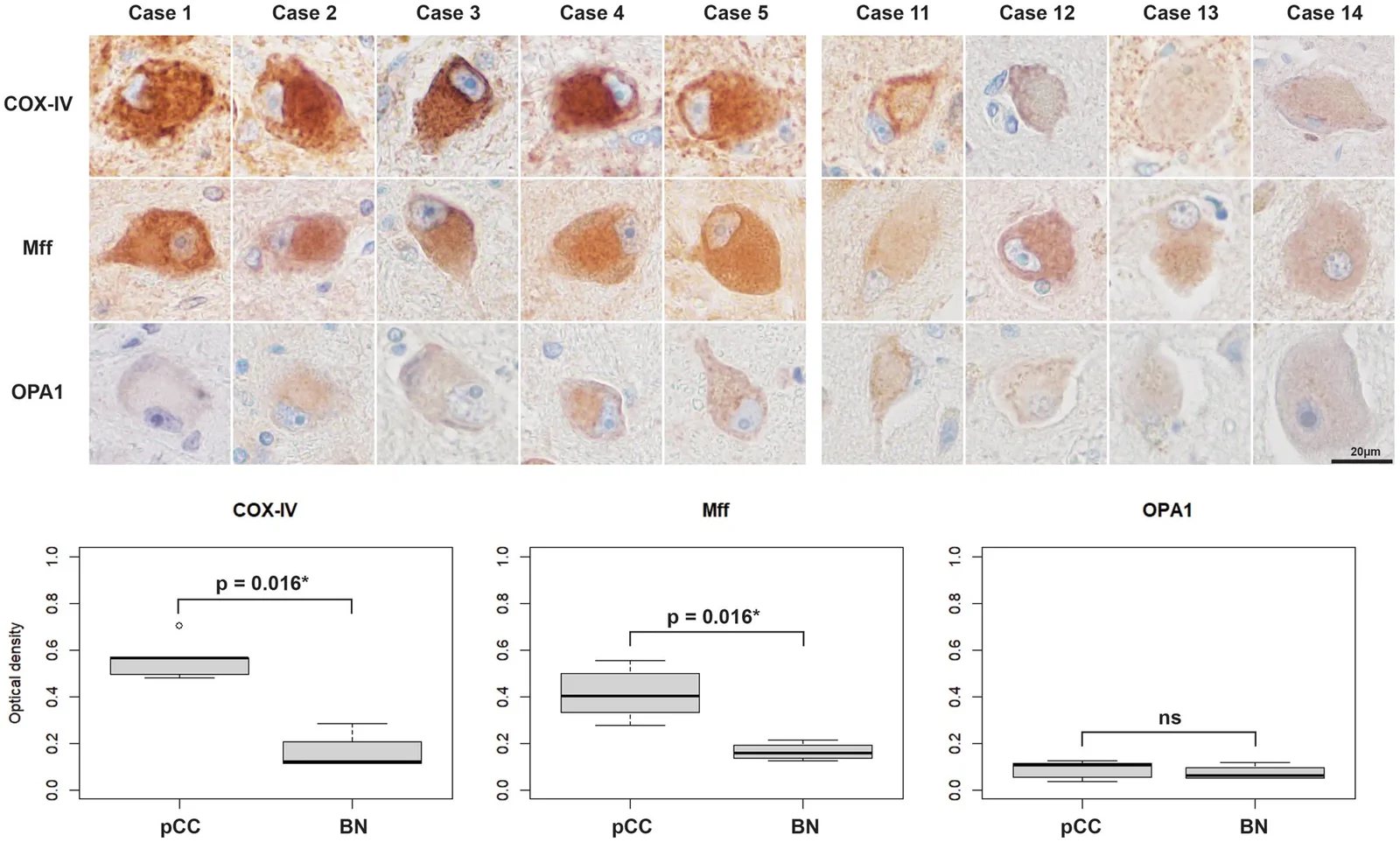
Comparison of immunohistochemical staining intensity for mitochondrial markers between pCC neurons and retrograde degenerated ballooned achromatic neurons. Morphometrical quantitative analysis of DAB staining optical density for COX-IV, Mff, and OPA1. Cell bodies with pellagrous CC exhibited significantly higher optical density for COX-IV and Mff compared to BNs (COX-IV: *P* = .016; Mff: *P* = .016; Mann–Whitney *U* test). Abbreviations: pCC, pellagrous central chromatolysis; BN, ballooned achromatic neurons.

In all 10 pellagra encephalopathy cases, the CC cells were negative for SMI-31, αΒ-crystallin, and all anti-tau antibodies examined (AT8, RD3, and RD4) (Table 3, Figure 2D, F-I, Figure 3A, C, D). SMI-32 immunoreactivity was weak or absent in the pellagrous CC (Table 3, Figures 2E and 3B). Ballooned achromatic neurons in the axonal injury case were positive for SMI-31, SMI-32, and αΒ-crystallin but negative for anti-tau antibodies (Table 3, Figure 3E-H). Ballooned achromatic neurons in the CBD cases were positive for SMI-31, SMI-32, and αΒ-crystallin, and moderately positive for AT8 and RD4, but negative for RD3 (Table 3, Figure 3I-L). AT8 immunostaining in CBD cases revealed that BN were less intensely stained than neurofibrillary tangles. In CBD cases, SMI-31, 32, and αΒ-crystallin-immunopositive cells were observed, particularly in the superior frontal gyrus. Ballooned achromatic neurons in the PiD cases were moderately positive for SMI-31, SMI-32, and αΒ-crystallin, and strongly positive for AT8 and RD3, but negative for RD4 (Table 3, Figure 3M-P). The number of SMI-31, 32, and αΒ-crystallin-positive cells in the PiD cases was lower than that of AT8- and RD3-positive cells. SMI-31, 32, and αΒ-crystallin-positive cells in the PiD cases tended to be observed in severely degenerated lesions around the hippocampus. Morphometrical quantitative analysis of DAB staining intensity for COX-IV, Mff, and OPA1 revealed that cell bodies with pellagrous CC exhibited significantly higher staining intensity for COX-IV and Mff compared to BNs (COX-IV: *P* = .016; Mff: *P* = .016; Mann–Whitney *U* test; Figure 4).

### Electron microscopy findings in CC cells

**Figure 5:**
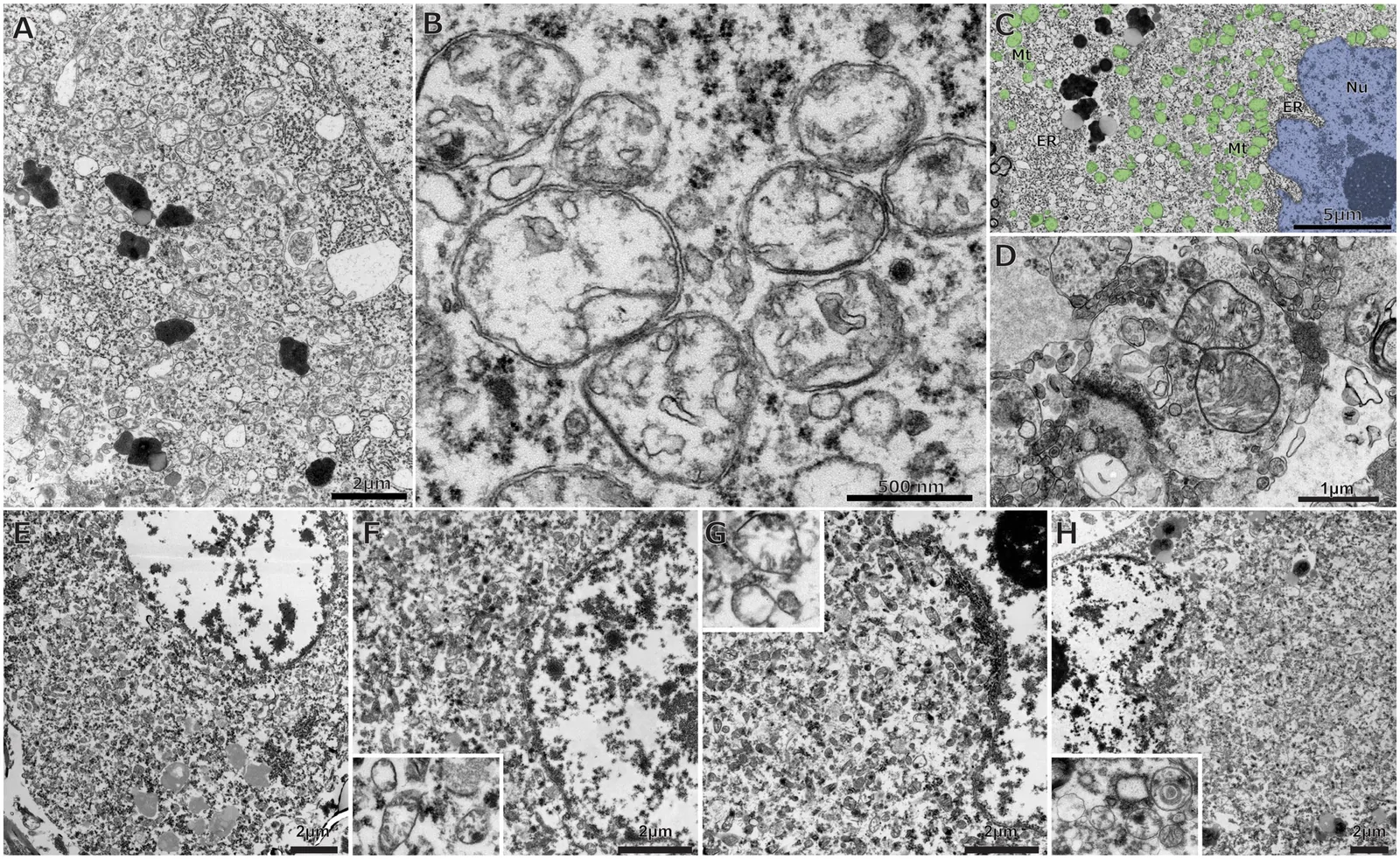
Ultrastructural findings in alcohol use disorder-related cases of pellagra encephalopathy: Case 1 to 5. (A-D) Electron microscopic analysis of Case 1. (E) Case 2, (F) Case 3, (G) Case 4, (H) Case 5. (A) A large number of small, round mitochondria were observed in the body of a central chromatolysis (CC) cell. (B) A high-magnification view of the mitochondria revealed reduced number of cristae, amorphous electron-dense precipitates, and their immature morphology. (C) A schematic representation illustrating the distribution of mitochondria (green) in relation to the nucleus (blue) and endoplasmic reticulum (ER). (Abbreviations: Mt, mitochondria; Nu, nucleus; ER, endoplasmic reticulum.) (D) In the presynaptic terminals within the same section, mitochondria with normally formed cristae are observed, unlike in CC cells. (E-H) Morphological changes in mitochondria, comprising a reduced number of cristae and a decrease in size, are observed to varying degrees. An increase in the number of mitochondria within the cell body and the presence of amorphous densities within the mitochondria are consistently observed.

Ultrastructural examination of the 5 pellagra encephalopathy cases revealed mitochondrial proliferation within neurons exhibiting CC, along with the presence of intramitochondrial amorphous densities. In some mitochondria, fragmentation into small spherical structures was observed, accompanied by a reduction in cristae. The severity of these morphological alterations varied significantly between cases; findings ranged from widespread cristae loss and fragmentation in nearly all mitochondria (Cases 1, 5; Figure 5A-C, and H) to cases where, despite increased mitochondrial density and amorphous densities, cristae were well-preserved and numerous barrel-shaped mitochondria were prominent (Cases 2, 3, 4; Figure 5E-G).

### Clinical and neuropathological findings in a representative case

**Figure 6:**
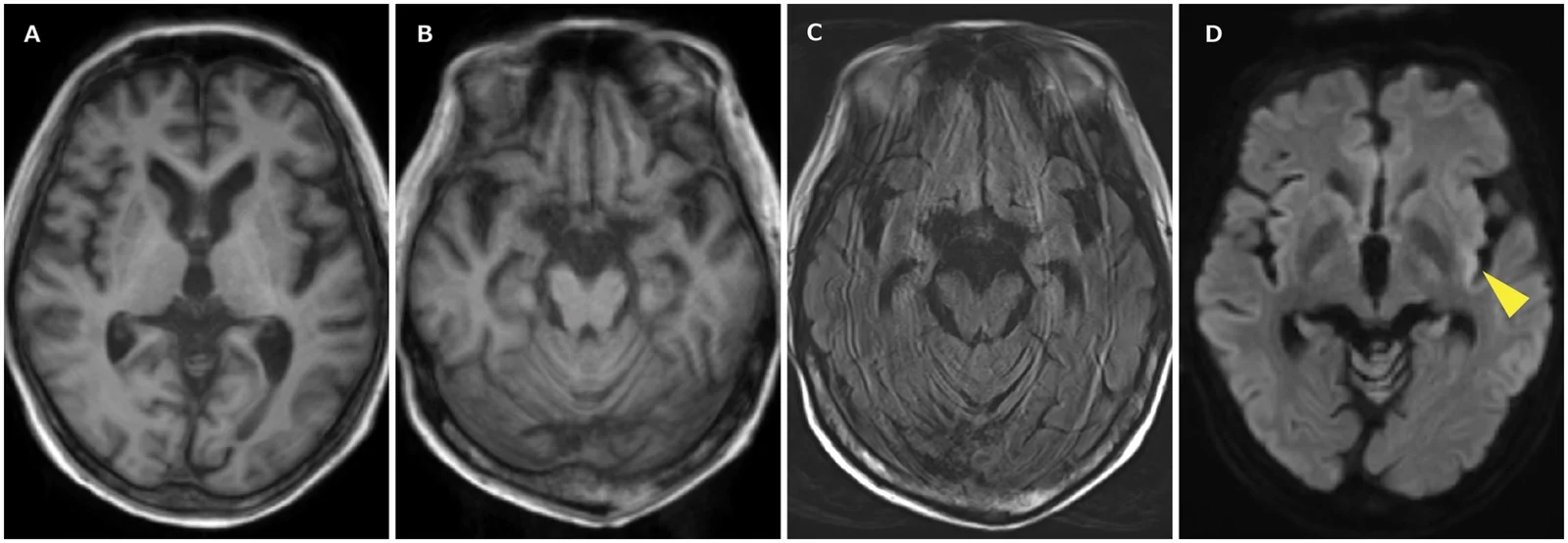
Brain MRI findings in a representative case of pellagra encephalopathy (Case 1). Brain MRI findings. Cerebral atrophy was not evident relative to the patient’s age on T1-weighted imaging (T1WI) (A), (B). A high-intensity area in the hippocampus was not evident on T2-FLAIR imaging (C). A mild high-intensity area was observed in the left caudal insula via diffusion-weighted imaging (DWI). This was considered a change due to an epileptic seizure (D).

Case 1: The patient was a male who died at 64 years of age. He had a medical history of treatment for pulmonary tuberculosis. His alcohol consumption increased after he left the workforce at approximately 43 years of age, and he was diagnosed with alcohol use disorder in his 50s. He was also diagnosed with epilepsy at the age of 52 years. At 63 years of age, his pulmonary tuberculosis recurred, and he was admitted to our institution for treatment. Blood tests on admission revealed a vitamin B1 level of 4.6 μg/dL and a vitamin B3 level of 5.1 μg/mL. On the day of admission, the patient was fully conscious. However, his level of consciousness gradually deteriorated after initiation of anti-tuberculosis medication, including isoniazid. Vitamin B supplements were administered at the start of his anti-tuberculosis treatment. During his admission, he experienced a prolonged decline in consciousness, intractable diarrhea, and skin pigmentation of his extremities. He experienced generalized convulsive seizures 3 times post-admission. An electroencephalogram immediately after the seizure revealed delta activity predominantly in the left parietal region. A brain MRI performed at the same time revealed a mildly increased signal intensity in the diffusion-weighted image of the left caudal insula (Figure 6A-D). Although his consciousness improved slightly after administration of anti-epileptic medication, he did not regain full consciousness during the interictal phase over several months. Markedly elevated liver enzymes were observed in the terminal phase. He died 4 months after admission from sepsis and multiple organ failure.

Post-mortem examination revealed that the brain weighed 1294 g. Macroscopically, atrophy of the cerebrum and cerebellum was not evident (Figure 2A). Microscopically, CC cells were found in the oculomotor nucleus, trigeminal spinal nucleus, gracile nucleus, and reticular formation of the medulla oblongata (Table 2, Figure 2C). The CC cells were negative for SMI-31, αΒ-crystallin, AT8, RD3, and RD4 (Figure 2D, F-I). CC cells were intensely immunopositive for COX-IV (Figure 2J). In this case, immunohistochemically, CC cells exhibited Mff staining intensity similar to that of non-chromatolytic cells (Figure 2K). In contrast, CC cells demonstrated weaker staining for OPA1 compared to the surrounding non-chromatolytic cells (Figure 2L). GFAP-positive reactive astrocytes were observed in the left insula (Figure S1), corresponding to the region of premortem postictal T2-weighted hyperintensity. Age-related neurodegenerative changes were minimal. Transmission electron microscopy revealed a marked increase in the number of small round mitochondria with reduced and immature cristae morphology in the neuronal cell bodies of CC cells (Figure 5A-C). Additionally, an increase in the number of free ribosomes was observed, while the rough endoplasmic reticulum was reduced and displaced to the periphery of the cell body. An increase in neurofilaments in the cell body was not evident. In the same sections used for the analysis of CC cells, mitochondria with preserved cristae were identified within presynaptic terminals and axons (Figure 5D). Based on these findings, this patient was pathologically diagnosed with pellagra encephalopathy.

## Discussion

This study revealed the following key findings. First, the pellagrous CC neurons in our study were frequently observed in the specific brain distribution consistent with previous study findings.^24^ This finding suggests that our pellagra encephalopathy cases were likely a population similar to those described in the previous study.^24^ Second, the pellagrous CC cells in our study were immunonegative for SMI-31, αΒ-crystallin, and the tau-related antibodies (AT8, RD3, and RD4), while retrograde degenerated neurons were immunopositive for SMI-31, SMI-32, αΒ-crystallin and ballooned achromatic neurons in CBD or PiD were immunopositive for SMI-31, SMI-32, αΒ-crystallin, and several tau-related antibodies. This finding suggests that the formation mechanism of pellagrous CC neurons is unrelated to cytoskeletal alterations and is distinct from that of ballooned achromatic neurons with other etiologies. Third, our immunohistochemical analysis of mitochondrial markers revealed that neurons exhibiting pellagrous CC possessed significantly higher immunoreactivity for COX-IV and Mff compared to BNs undergoing retrograde degeneration. Especially, our detailed neuropathological analysis for the representative case revealed that the pellagrous CC cells in this case had lower intensity of immunoreactivity for OPA1 which is associated with mitochondrial cristae maintenance, while COX-IV and Mff immunoreactivity was preserved. Furthermore, ultrastructural examination revealed that the cytoplasm of pellagrous CC neurons contained numerous mitochondria and intramitochondrial amorphous densities. Additionally, some of these mitochondria exhibited fragmentation and disorganized cristae, particularly in Case 1, a representative case. In pellagrous CC, the variability in cristae disorganization observed under electron microscopy is consistent with the variability in OPA1 staining intensity, which showed no statistically significant reduction relative to BNs. These findings suggest that neuronal energy deficits resulting from NAD^+^ deficiency postulated in pellagra encephalopathy may induce mitochondrial fragmentation. Fourth, our pellagra encephalopathy patients included not only alcohol use disorders but also those with impaired oral intake due to severe physical illness or progressive neurodegenerative disease. In addition, none of our cases presented with all classic “4Ds.” These findings reveal the diversity of patient backgrounds and clinical manifestations in pellagra encephalopathy. Furthermore, our findings suggest that pellagra encephalopathy can occur in patients with low physical reserve, such as those with severe systemic diseases or end-stage neurodegenerative diseases, although it can worsen their prognosis by causing impaired consciousness.

### Mitochondrial alterations in pellagra encephalopathy-associated CC

The majority of the energy required by neurons is supplied by mitochondria.^30^ To maintain cellular homeostasis, mitochondria actively change their shape via fusion, fission, mitophagy, and transport.^31^ Mitochondrial fragmentation is a morphological change induced by environmental stressors surrounding the cell. This process is characterized by mitochondrial fission^19^ and alteration of the cristae structure.^20^^,^^21^^,^^23^ Mitochondrial fragmentation results from mitochondrial depolarization^20^^,^^21^ and impairment of NAD^+^ synthesis.^16^ Furthermore, an increased intracellular ADP/ATP ratio is known to downregulate mitochondrial transport in axons.^32^

**Figure 7:**
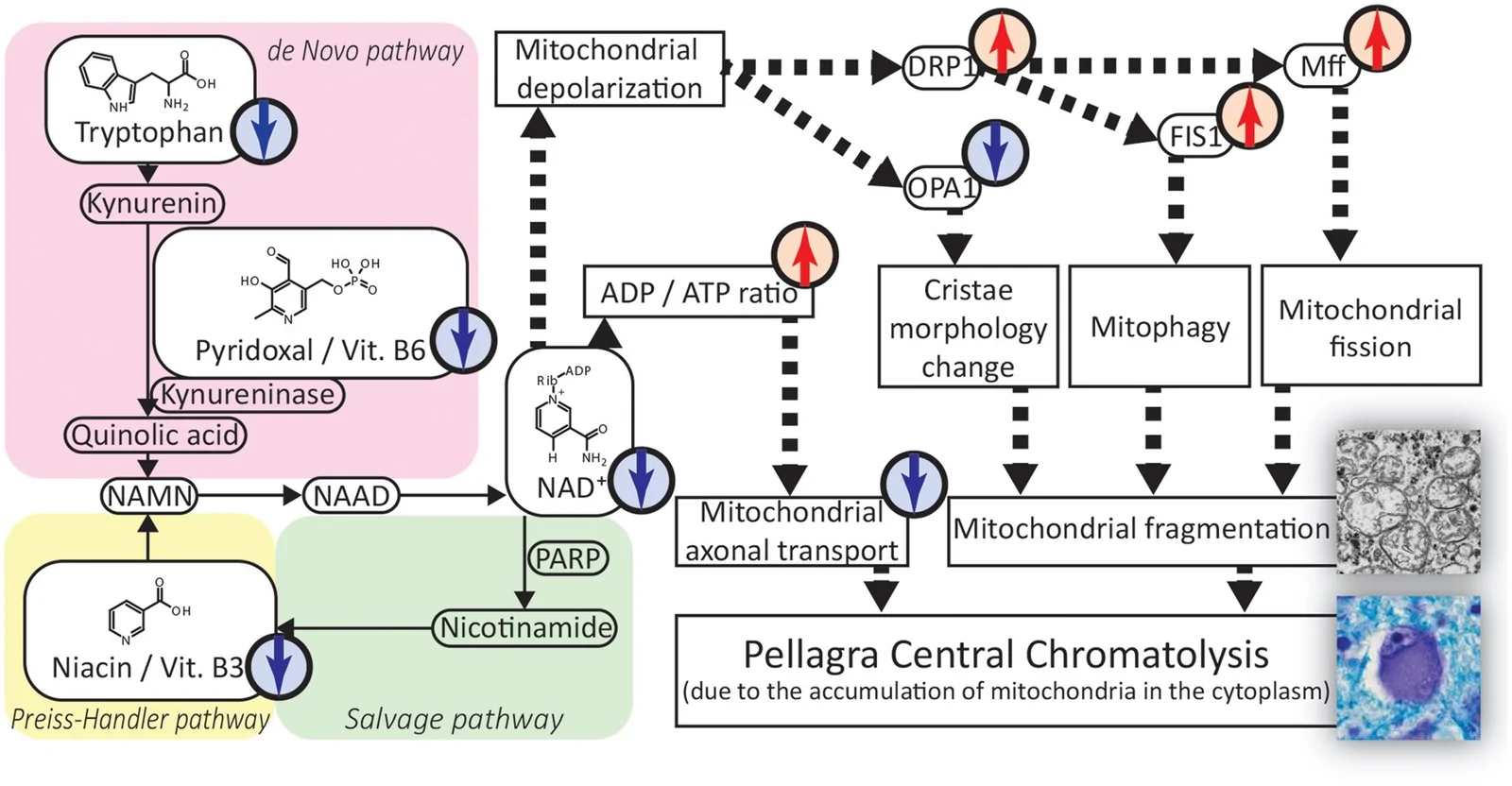
A proposed pathomechanism for pellagra-associated central chromatolysis (CC) based on previous literature. This schematic diagram illustrates that nicotinamide adenine dinucleotide (NAD^+^) deficiency, which is thought to be induced by deficiencies in vitamin B3, tryptophan, and vitamin B6, is associated with mitochondrial reactions that can lead to CC. Skeletal formulas were created using ACD/ChemSketch (ACD/Labs, Inc.). Abbreviations: Vit. B3, vitamin B3; Vit. B6, vitamin B6; Rib, ribose ring.

Impairment of NAD^+^ synthesis can also result from a deficiency of vitamin B3, a substrate of the Preiss–Handler pathway, and vitamin B6, a cofactor in the tryptophan–kynurenic acid pathway. Furthermore, mitochondrial morphological alterations are hypothesized to result from NAD^+^ deficiency in pellagra encephalopathy. Immunohistochemical analysis of COX-IV and Mff demonstrated mitochondrial proliferation in neurons exhibiting pellagrous CC (Figure 4). In the representative case, CC cells were intensely immunopositive for COX-IV (Figure 2J) and Mff (Figure 2K), but reduced OPA1 immunoreactivity (Figure 2L), compared to non-chromatolytic cells. Ultrastructural findings revealed an accumulation of small, round mitochondria with intramitochondrial amorphous densities and disorganized cristae in the cytoplasm. Intramitochondrial amorphous densities have been reported as a characteristic finding in injured cells, including those affected by ischemic insult.^33^ These findings support our hypothesis, based on the previous literature (Figure 7), that NAD^+^ deficiency and insufficient cellular energy status in pellagra encephalopathy can cause mitochondrial fragmentation and the accumulation of mitochondria in the neuronal cell body. Mitochondrial fragmentation is thought to represent a transitional period in the process of cell death. The administration of vitamin B3, which is expected to restore cellular NAD^+^ levels, may halt the process of cell death in pellagra encephalopathy. This is consistent with the clinical observation that symptoms of pellagra encephalopathy resolve with vitamin B3 treatment in clinical settings.

In the present study, pellagrous CC cells were predominantly found in the precentral gyrus, oculomotor nucleus, raphe nucleus, pontine nucleus, pedunculopontine nucleus, trigeminal spinal nucleus, cuneate nucleus, gracile nucleus, arcuate nucleus, and reticular formation of the pons and medulla oblongata. The distribution pattern of pellagra CC cells is consistent with findings from a previous neuropathological study of pellagra encephalopathy.^24^ The preferential involvement of these specific anatomical regions, which remains consistent across cases, not only suggests that our patients are comparable to previously reported populations of pellagra encephalopathy but also implies the possibility of regional differences in vulnerability to NAD^+^ deficiency.

### Differences between pellagrous CC cells and ballooned achromatic neurons in retrograde degeneration or neurodegenerative diseases

The characteristics of retrograde degeneration in neurons with axonal injury have been well-established. Previously reported observations of motor neurons in animal studies of spinal cord anterior root transection revealed an increase in total protein and RNA in the cell bodies of axotomized motor neurons, with almost all of the increased protein being in the cytoskeleton.^34^ A study of axotomy experiments in which immunohistochemical evaluation was performed using various neurofilament antibodies revealed that after transection, the cell body showed a marked enhancement of neurofilament immunolabeling, while axonal transport was reduced and the axonal diameter proximal to the transection site was decreased.^35^ These findings are interpreted as an increase in neurofilaments in the cell body and a block in their translocation to the axon. BN observed in neurodegenerative diseases such as CBD and PiD were also labelled with neurofilament antibodies, suggesting a mechanism similar to retrograde degeneration in axonal injury.^26^

In contrast, a previous immunohistochemical study in CC cells with pellagra encephalopathy reported weak immunolabeling with anti-phosphorylated neurofilament antibodies.^36^ In the present study, pellagrous CC cells were not immunohistochemically labelled with anti-phosphorylated neurofilament antibodies or with any of the anti-tau antibodies. This result differs from the aforementioned retrograde changes observed in neurons with axonal injury, as well as BN in the CBD and PiD. The immunohistochemical features of CC cells in pellagra encephalopathy observed in the present study were consistent with previous reports.^36^

Previous ultrastructural studies on retrograde degeneration in axonal injury reported increases in neurofilaments, free ribosomes, and mitochondria in the cell bodies.^37^^,^^38^ On the other hand, in a few case reports, electron microscopic examination of pellagrous CC cells revealed a marked increase in small mitochondria and a mild increase in free ribosomes but no increase in neurofilaments.^39^^,^^40^ The electron microscopic findings in our study were also characterized by numerous mitochondria with amorphous densities, an increase in free ribosomes, and inconspicuous neurofilaments. These findings were consistent with previously reported electron microscopy images of pellagrous CC cells. Therefore, CC cells in pellagra encephalopathy can be distinguished from neurons undergoing retrograde degeneration due to axonal injury on the basis of electron microscopic features, particularly neurofilaments and mitochondrial morphology.

Taken together, these immunohistochemical and electron microscopic findings suggest that CC cells in pellagra encephalopathy have a different pathological mechanism of lesion formation from those involved in axonal injury and neurodegenerative diseases such as CBD and PiD.

### Clinical background of the pellagra encephalopathy patients

The 10 pellagra encephalopathy cases examined in this study included 5 with a history of alcohol use disorder. This finding is consistent with the known risk factors for pellagra encephalopathy in developed countries. Additionally, the study included cases in which oral intake was impaired due to progressive neurodegenerative disease or severe physical illness. Moreover, isoniazid was administered in Case 1, which was presumed to be a contributing factor to pellagra in this patient. Previous studies have suggested that elderly individuals in long-term hospital care may be at risk of vitamin deficiencies^41^ and have also indicated a relationship between low vitamin B3 intake and cognitive decline in elderly individuals.^42^^,^^43^ Therefore, elderly individuals and long-term care patients may be at risk of developing pellagra encephalopathy. Pellagra encephalopathy often presents with disturbance of consciousness. Although it is frequently associated with alcohol use disorder cases and anorexia nervosa cases, it can also occur in other vulnerable populations, such as the elderly or patients with severe physical illness. Moreover, pellagra encephalopathy may further worsen the prognosis of these patients. Therefore, close clinical observation and timely vitamin administration are important for preventing the development of pellagra encephalopathy.

### Limitations

This study has the following limitations. First, our detailed clinicopathological analysis is focused on a single case (Case 1), whose impaired consciousness could have been influenced by several confounding factors. For instance, the lack of full recovery in his conscious state over several months of the interictal phase might be attributed to non-convulsive status epilepticus. Moreover, pellagra encephalopathy itself may also exacerbate epilepsy.^3^

A further limitation relates to molecular and genetic analysis. Fresh frozen brain samples were unavailable for genetic or biochemical analysis. Therefore, it was not possible to analyze gene expression related to mitochondrial alterations in affected regions. Consequently, our discussion of the mitochondrial response in pellagra encephalopathy is based on an interpretation of previous literature, rather than on gene expression data directly derived from the sample of the pellagra encephalopathy cases.

The number of axonal injury cases in the present immunohistochemical study was limited. This is due to the limited time window in which achromatic neurons appear following axonal injury secondary to cerebral trauma, and only one case in our institution’s archive contained observable achromatic neurons suitable for investigation. Our findings in this case of axonal injury are consistent with those of previous studies on retrograde degeneration following axonal injury.^35^^,^^36^

For electron microscopic observations, the samples were fixed with formaldehyde and later refixed with osmium. This fixation process, combined with potential sample deterioration over time, may have introduced artifacts. Therefore, it cannot be ruled out that the observed mitochondrial membrane structures were unintentionally and artificially altered. Furthermore, the number of cases evaluated by electron microscopy is limited due to sample availability.

## Conclusion

The pathomechanism of CC cells in pellagra encephalopathy likely involves mitochondrial alterations and neuronal energy deficit resulting from NAD^+^ deficiency postulated in pellagra encephalopathy. This mechanism is fundamentally distinct from the retrograde reactions observed in axonal injury or ballooned cells observed in neurodegenerative diseases. Our findings suggest that neurological deficits can arise from primary cellular metabolic alterations, not solely from mechanical trauma or neurodegenerative diseases.

In developed countries today, pellagra encephalopathy is considered a rare disease and occurs only under limited conditions. However, without proper assessment, diagnosis, and treatment, it can be fatal. Pellagra encephalopathy is not only a concern for patients with a history of chronic alcohol use disorder or anorexia nervosa, but also for patients with oral intake difficulties due to severe physical illness and those receiving medications such as antituberculosis drugs. Pellagra encephalopathy remains an important differential diagnosis in patients with prolonged impairment of consciousness.

By investigating a larger population of human pellagra encephalopathy cases than previously reported,^36^ we provide a robust immunohistochemical comparison between pellagrous CC neurons and ballooned achromatic neurons of other etiologies. Furthermore, to the best of our knowledge, this study is the first to show that multiple cases of pellagra encephalopathy—diagnosed based on clinical history, lesion distribution, and the absence of cytoskeletal markers—consistently exhibit elevated mitochondrial proliferation markers and fragmentation, as evidenced by immunohistochemistry and electron microscopy.

Further case accumulation and studies are required to elucidate the relationship between clinical manifestations, such as the presence or absence of impaired consciousness, and the histopathological distribution of pellagrous CC lesions.

## Supplementary Material

nlag058_Supplementary_Data

## Data Availability

The data that support the findings of this study are available from the corresponding author upon reasonable request.
