# Supplementary material for: Mitochondrial alterations in pellagra-associated central chromatolysis: Comparison with ballooned achromatic neurons of other etiologies
Source: J Neuropathol Exp Neurol. 2026 Aug 6;85(10):1104–16. doi: 10.1093/jnen/nlag058 (PMC13612925; doi:10.1093/jnen/nlag058)
Supplement: Supplementary file 1 [file nlag058_supplementary_data.zip › Supplemental figure1.pdf]

Supplemental figure 1. GFAP-positive reactive astrocytes on left insula: Case 1.

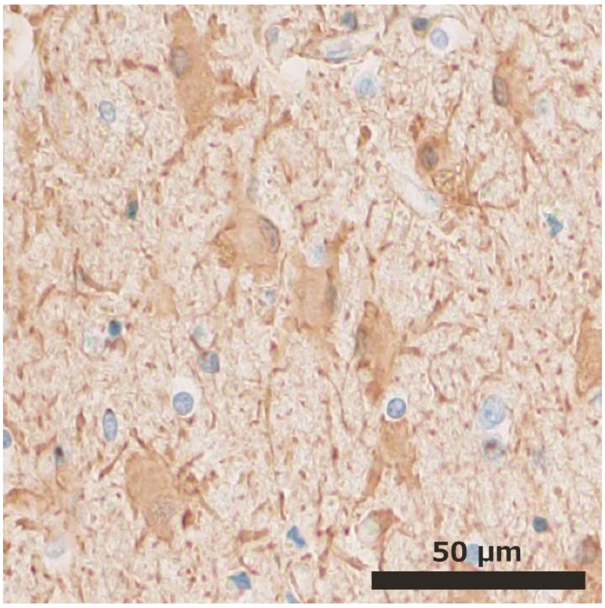

Astrogliosis in the left insula characterized by GFAP-positive reactive astrocytes. This finding is located in the area of premortem postictal T2-weighted hyperintensity.
